# Food and the Teeth

**Published:** 1866-10

**Authors:** L. C. Ingersoll

**Affiliations:** Keokuk, Iowa


### FOOD AND THE TEETH.

BY DR. L. C. INGERSOLL, KEOKUK, IOWA.

Read before the Iowa State Dental Society.

THIS is the subject of an article, ably written, and published, several years since, in the medical journals of the country, and recently republished in our dental journals. The writer, in his very clear observations on the constituents of the food of children, as connected with the decay of the teeth, and the physical constitution of women in America, has justly made his theme one of paramount importance.

As it introduces facts and principles so important in dental science and so seldom considered by Dentists; I shall make it a basis of some general remarks.

First, let me premise, the gateway to this field of investigation has been quite blockaded by prejudice. Nor is this obstruction, by any means, yet wholly removed. How far the profession of medicine are responsible for this prejudice, it is not needful to determine. Among all reforms, the dietetic reforms has seemed to be the butt of ridicule; assuming at once, that *taste* alone, as an instinct of animal nature is a faithful minister to appetite, and that taste and appetite, acting harmoniously, need as little of human guidance in supporting nutrition as do the heart and lungs in their involuntary action. The study of medicine looking to pathological, has seemed to overlook physiological conditions, and to place health and hygiene without the pale of its immediate observation. The theory of medicine has led to the study of the incompatibility of pathological conditions, thereby to antidote disease with disease, forgetting that the natural antagonism of pathological conditions is a physiological condition, and that physiological action is a perpetual antidote to disease. It matters not how this prejudice has been imbibed, but it has been well nigh universal against any effort to accomplish permanent hygienic results by prescribing courses of diet. Medicine has been the God of Medical science, and worshipped by all the people. There has, however, crept in, of late years, through the channel of medical and dietetic reforms, a wholesome measure of infidelity concerning this worship. Now, the more natural ways of producing physiological results are looked upon with favor. Chemical compounds are reduced, and decoctions are strengthened with medical advice.

Dr. Sylvester Graham, who thirty years ago was only laughed at, has gained esculapian honors, and his name has been rendered immortal by giving to the world the staff of life, inscribed with his own name, "Graham bread."

If the fact which science has established, shall be made plainly evident to us all, that Graham bread contains more of the constituent elements of bone and dentine than any other article of daily consumption, we, as dentists, will have occasion to ascribe to Sylvester Graham greater honors than we claim for ourselves, because he offers to mankind the ounce of prevention, while we offer the pound of cure.

The subject of the relations of food to the teeth is one which needs a thorough canvassing, and a consideration and elucidation which I am at present unable to give. Topics bordering upon it have been investigated and discussed at length; and the discussions have verged so closely upon the facts which contained the real issue, that the greater is the wonder that the kernel was not reached after such a cracking of the shell.

These two questions compass the whole subject:

First.—Does food sustain such a relation to the teeth that diet has a controlling influence in their organization, causing the teeth to be well or poorly organized as one or another kind or class of articles of food is taken for nourishment?

Second.—Does food sustain such a relation to the teeth that in case they are developed in a frail and poorly organized condition, they may, when the cause ceases, by a proper course of diet, be so changed in their elemental composition as to render them less subject to caries—thus counteracting wholly, or in great part, the early tendency to decay?

In order not to complicate the question needlessly, it must be assumed that the functions of nature act their part in a normal manner.

Again and again have we turned back to hereditary causes —to functional derangements of the parent transmitted to the child. Here we found facts so abundant as to seem at first view, to settle fully the question of dental organs in ruin. We contentedly fall back upon an idiosyncrasy. Back of all this, however, there is a cause of this so called idiosyncrasy. The human body is not a machine which when made and put in motion needs no further re-making. It is a machine of functional powers, which, in their action, are continually reproducing the whole and all its parts specifically. This body, originally made of the "dust of the earth," is still made of *dust*. Especially significant is this language in reference to the elemental structure of the bony framework of the human system.

That very comprehensive term, *nutritive*, names the whole class of functional powers by which this perpetual reconstruction of bone-dentine, cartilage, nerve-tendon, membrane, muscle, and every other part of the human body, is accomplished. Yet, in the general use of this term, the bony structures, especially the teeth, are left almost entirely out of the account. This is evident, first, from the division so generally made of nutritive elements into *nitrogenous*, and *non-nitrogenous*—flesh-producing and fat-producing. It is evident, again, from the analyses so often given of various articles of food, in which the definite proportions of sugar, starch, gluten, caseine, etc., are given, and account is taken of their value for the nutrition of the soft parts, while scarcely mention is made of the mineral elements contained in these various articles of food, which serve to build up the framework of the human body, for a support to all the soft tissues composing the organic structure of more vital parts.

So far as the teaching of most writers on this subject, whom I have read, goes, the human body might be but a mass of animal jelly; for mineral elements are seldom, if ever, mentioned in connection with nutrition. While the bony structure is not wholly ignored as a substantial part of the human body, the method of its existence, growth and development is seldom brought to our notice. Writers seem to assume its existence as a framework of the human body, put together bone to bone, as an architectural gardner frames and sets up an arbor in his grounds, and that the flesh duly nourished, comes upon the bones, just as plants and vines cover the frame-work of an arbor, by successive years of nourishment and growth. But let us not forget that skeleton bodies, unlike a skeleton arbor, grow by the assimilation of food, as do the soft parts—that a human skeleton is a living thing, tooth and bone—and needs nourishment to support its life.

Suffer me now to go back a little and modify my previous statement. The mineral elements of human growth have not been *entirely* ignored. For, under certain circumstances, the demand has been acknowledged. The claim of the bony structure for mineral has been looked upon as an occasional, rather than as a perpetual necessity to supply a perpetual waste.

About the year 1856, the Materia Medica was supplied with a new article called "Chemical Food," which was chiefly a solution of phosphates, to be taken into the stomach as food for the bones.

Von Pleufer, the most renowned physician of Munich, induced the apothecaries of his city to keep for sale, a soup largely impregnated with bicarbonate of potash. Others have suggested lime-water, soda-water, ash-water, besides various mineral spring waters. hoping to derive from one, or all of them, the solid earthy elements of human bone, including the teeth.

So far as these simple elements and chemical compounds are designed for infants, deprived, for any cause, of natures provision for their nutriment—the mother's milk—at that early age, when milk is the *only* suitable nourishment they are a suggestion of thoughtful wisdom. Though it must be borne in mind that the best *substitute* is but a poor imitation of human milk, which is nature's only perfect food.

But I must be allowed to say that, so far as these mineral solutions and compounds are proposed for supplying the embryo child through the maternal organism, or for meeting the demands of children or adults after the development of the teeth, the proposition indicates a misconception of, *not* nature's demands, but of nature's provision for supplying these demands, by the use of natural food, organically combining the constituent elements of growth. A consistent application of the theory of chemical food might require that all food be reduced chemically to its constituent elements, and an artificial mode instituted for producing flesh and bone, while the assimulative functions should lie dormant.

What, let me ask, is the physiological indication of the early development of the teeth? I answer, the indication is, that the infant organism is, at that period, ready for the reception of more solid food than milk; that henceforward, from solid substances are to be derived a large proportion of the elements of nutrition. Here we arrive at a fact which must never be lost sight of in the consideration of the subject of nutrition,—namely, that from a vast variety of natural productions designed by Providence—not by the chemist—for human food, man must derive *every element* of nutriment for the body—the whole body—for the bone and dentine as well as for muscle and fat.

When men talk of nutrition and growth, how often does it appear, from the language used, that only the production of flesh and fat is intended! In an ably written article in the last report of the Commissioner of Agriculture, the writer, in comparing various articles of food adapted both to man and to domestic animals, says:—"If one plant produces thirty per cent. more than another of flesh-producing or fat-producing—nitrogenous or non-nitrogenous substance, it is manifest that the one richest in fat-producing or flesh-producing, should be selected." "Peas and beans, and other leguminous plants," he says, "derive their superior nutritive value from the large proportion of nitrogenous, or flesh-producing material which they contain." He makes no mention of the large proportion of lime which these contain. Why are beans so valuable an article of food for the sheep? I answer, because they nourish so well the firm, horny fiber of his fleece, as well as his body. And why does the sheep thrive best on rocky soil, and seek the shortest pasture, but that such soil and such pasture is richest in silicic, phosphoric, carbonic, and sulphuric acids, holding in solution the mineral portion of the soil, out of which is this growth of turf? Reports tell us of sheep thriving in flocks of thousands, on the sand-barrens of Australia, licking the dust beneath their feet, where but a trace of dried vegetable substance can be seen.

Turn to a swine, who considers it his "sole duty to grow fat," and ask for his choice of food, and he will cry *corn* every time, because that is richest of all grains in the non-nitrogenous, fat-producing substances. In selecting a swine to feed for home consumption, you do not search for a large-boned animal, but rather for one small and slender boned, with a frame-work only sufficient to support the adipose matter with which you intend he shall be abundantly clothed. Flesh and fat are all you wish to grow. But when you are looking for a horse having strength, vigor and endurance, you look for a well developed, bony structure, as well as for a firm, compact muscle. Corn-fed colts are wanting here; and, when over-grained, they will greedily gnaw the fences and bark of trees for a supply of mineral, and will turn with loathing from rich food to the ground and lick the dust from the street. Physiological science is interested to reduce to a practical principle the adage, "every man must eat his peck of dirt;" and the application is common both to men and animals.

The writer last quoted makes another statement concerning the relative values of wheat, oats and barley. He says that the latter two are less valuable than wheat, because they contain less gluten, and more starch, as though these were the only important elements for the growth of the body. Look at the sturdy Scotchman living upon his barley-bread and oat-meal porridge, and you see a denial of this statement in his wiry muscle and compact frame.

I wish now, with the foregoing remarks before us, to come directly to the point, that the *teeth* grow by nourishment as do other parts of the body, that the nutrient elements for both the soft and the solid parts of the human body are found in the food that God has adapted to the physical nature of man—that for the purpose of supplying the demand for the daily nutrition of every part, vegetable and animal substances designed for food are possessed in due proportion of the ultimate elements of a well organized human body—that there is no demand for *chemical food* but for *natural food* in a complete organization of bone and dentine.

These varied propositions contain my views of the method of developing and continuing a perfect organization of human teeth.

To make the subject more clear let me refer you to the laboratory of the human body, the stomach and the alimentary canal. Into the stomach, food in the crude mass, is received. Here it is reduced to a pulpy consistency, its elements put in solution for a thorough chemical analysis while passing the long and devious line of the intestinal tube. The multitude of absorbants, with most persistent eagerness, and with a kind of elective affinity unknown to human science, take up the atomic elements of bone, dentine, muscle, fat, according to their specific functions, and with these elemental parts of every part near and remote, freight the arterial system, which, like canals and rivers running every whither deposit their material for growth, or for repairing organic or inorganic waste.

The fact of a perpetual waste of elemental particles, and a corresponding supply of the waste, is, in some particulars, plainly evident. We see, for example, the hair and nails cut off, and the whole renewed as before. We see the waste of the cuticle perpetually supplied. We see abrasions of the skin, and deeper wounds, in a few days or weeks, thoroughly repaired. These facts serve to illustrate and declare the principle. Nor is it wholly inferential from these data that the same economy of nature obtains throughout the system.

If there be such waste (and it is estimated that in about seven years, more or less, the whole body has gone through with a change of its elemental parts) then it is evident that there must be a corresponding supply, by the nutritive functions of this complete waste.

The fact that elements which enter in as component parts of, or are mixed with food, enter into the bony structure, is proved by experiment. A pig, previous to being killed, had all his food colored with madder. After killing, the bones of the animal were distinctly seen to be colored red. The teeth were not included in the examination which was made.

While this theory of waste and supply is believed, concerning the soft parts, it is not so readily accepted concerning the dentine.

If it can be established concerning the dental organs, we have arrived at *the* all important fact in determining the method of securing and perpetuating a healthy and enduring organization of the teeth.

I shall not be foolish enough to think that I can demonstrate this so fully to your comprehension as to admit of no doubt; although I believe the proposition. The most I can do is to suggest some trains of argument which, if they could be accompanied with proper research, and experiment would carry, to most minds, so full a probability of fact, as to induce an almost willingness to close the argument with those initial letters, which so often conclude the solutions of mathematical problems, *Q. E. D.*, *quod erat demonstrandum.*

First, I reason from analogy: If the system of waste and supply pertains to the fluids of the body; to the semi-fluids and adipose matter ; to the membranous and fibrous portions; to tendons, cartilage, hair and nails, the natural inference is that waste and nutrition pertain as a principle of life and growth, to the yet firmer structures, bone and dentine.

Again, the nutritive function is vitalized with the life force, as truly as is any other bodily function ; and we have no reason to suppose that it loses this vital force, at any point in the system, or that its functional power ceases anywhere sooner than any other vitalized function. That by the action of the nutritive system, the bones and the teeth come to a maturity corresponding to the growth and maturity of other parts of the body, all agree. Having now brought to maturity the dental organs, does the nutritive function by which they were produced cease its power and existence? Is this nature's mode of completing her work? Such a cessation of function would be death. The continued existence of the nutritive function after bodily maturity and the necessary exercise of the function is admitted as concerns the soft parts. What reason, then, to suppose the cessation of the nutritive function as regards the teeth, after their maturity? Again, Philosophy teaches that the existence of anything presupposes a purpose in its existence, and a use in its relation to other things. If dentine were a dense impermeable solid, like diamond, we should be compelled to arrive at conclusions which would accord with its nature. But when we find it of a tubular structure—the tubules diverging from a longitudinal central cavity into which they open, and when we find this central cavity occupied by a vascular, sensitive organ, which we call the pulp, the earliest moment of our discovery prompts the inquiry, what office have these dentinal tubes to perform in relation to this central organ? Their existence, and their tubular structure indicate *some* related function. With microscopic eyes we see more closely the organization, and discover a lining membrane, and a circulating fluid in each tubule. This identifies the relationship to the vitalized central power. Tell me now the office work and functional powers of the dental pulp, and I will tell you the functional powers of these related organs. If you answer, *life* and *nutrition*, I answer *life* and *nutrition*. Life and nutrition are correlative terms. Life makes nutrition a necessity; and nutrition presupposes life. We must conclude, therefore, that the dentine is possessed of a living, nutritive power. But nutrition implies a waste of dentine, corresponding exactly in amount with the nutritive element supplied. In this action of the nutritive principle we have all we can ask in securing a firm dental structure. In accomplishing it, with an ordinarily healthy tone of bodily functions, we have but to select such articles of animal and vegetable food as contain a due proportion of the mineral elements of which the teeth are chiefly composed. Thus they will be built up, thoroughly dentified and enameled, to endure till the organic nature, to which they minister, shall no longer need the mastication of food in the support of life.

